# FOXO3 induces ubiquitylation of AKT through MUL1 regulation

**DOI:** 10.18632/oncotarget.22793

**Published:** 2017-11-30

**Authors:** Sun-Yong Kim, Hyo Jeong Kim, Hyung Kwon Byeon, Dae Ho Kim, Chul-Ho Kim

**Affiliations:** ^1^ Department of Otolaryngology, Ajou University School of Medicine, Suwon, Republic of Korea; ^2^ Department of Molecular Science and Technology, Ajou University, Suwon, Republic of Korea; ^3^ Department of Radiology, Yonsei University College of Medicine, Seoul, Republic of Korea; ^4^ Research Institute of Radiological Science, Yonsei University College of Medicine, Seoul, Republic of Korea

**Keywords:** FOXO3, AKT, MUL1/MULAN/GIDE, ubiquitylation, cisplatin

## Abstract

AKT (also known as protein kinase B, PKB) plays an important role in cell survival or tumor progression. For these reasons, AKT is an emerging target for cancer therapeutics. Previously our studies showed that mitochondrial E3 ubiquitin protein ligase 1 (MUL1, also known as MULAN/GIDE/MAPL) is suppressed in head and neck cancer (HNC) and acts as negative regulator against AKT. However, the MUL1 regulatory mechanisms remain largely unknown. Here we report that cisplatin (CDDP) induces thyroid cancer cell death through MUL1-AKT axis. Specifically, CDDP-induced MUL1 leads to ubiquitylation of active form of AKT. We also observed that the role of forkhead box O3 (FOXO3) is pivotal in CDDP-induced MUL1 regulation. FOXO3 knock-downed cells show resistance against CDDP-mediated MUL1-AKT axis. CDDP-mediated intracellular ROS increment plays an important role in FOXO3-MUL1-AKT signal pathway. The data provide compelling evidence to support the idea that the regulation of FOXO3-MUL1-AKT axis can be a novel strategy for the treatment of HNC with CDDP.

## INTRODUCTION

AKT (also known as protein kinase B, PKB), a well-known serine/threonine protein kinase [1], plays an important role in cell survival [2] or tumor development. Genetic mutations and amplifications confer hyperactivation of AKT in human solid tumors and hematological malignancies. Inactivating mutations or loss-of heterozygosity in tumor suppressor genes lead to hyperactivation of AKT [3–5]. The somatic mutation of *AKT1* isoform associated with the substitution of glutamic acid by a lysine at amino acid 17 (E17K) of AKT1 has been reported in human breast, colorectal, ovarian cancers, and lung squamous cell carcinoma [6, 7]. Separate studies agree that AKT is hyperactivated in liver [8], lung [9, 10], colon [11], bile duct [12], pancreas [13–15], and head and neck cancer (HNC) [16, 17].

Several studies provided critical information of AKT signaling regulation by identification of regulators such as phosphatidylinositol-3-kinase (PI3K) or phosphatase and tensin homolog (PTEN) [18]. PTEN is among the most frequently mutated tumor suppressors [19]. Negative regulators such as C1 domain-containing PTEN, carboxy-terminal modulator protein (CTMP), TRB3, Keratin K10 and PH domain leucine-rich repeat protein phosphatase (PHLPP) are also reported to inactivate AKT [20–24].

Protein digestion by the ubiquitin-proteasome system (UPS) is crucial in many cellular processes [25]. In particular, lysine 48 (K48)-linked polyubiquitin-protein conjugates are recognized and destroyed by the 26S proteasome. Several ubquitin (Ub) E3 ligases have been described as responsible for incorporating K48-linked ubiquitylation of AKT [26]: E3 ligases carboxyl terminus of Hsc70-interacting protein (CHIP) [27], breast cancer early-onset 1 (BRCA1) [28], tratrico-peptide repeat domain 3 (TTC3) [29], and mitochondrial E3 ubiquitin protein ligase 1 (MUL1) [30].

MUL1 has been identified as a AKT negative regulator through induction of K48-linked ubiquitylation at K284 residue [30]. Previously, we reported that MUL1 is suppressed in HNC and contributes to cancer development [31]. MUL1 may act as a tumor suppressor protein in some cancers however, MUL1 regulating signal pathways remain unclear.

Forkhead box (FOX) proteins are a superfamily of evolutionarily conserved transcriptional factors which play important roles in a wide variety of cellular processes such as proliferation, cell cycle arrest (e.g., p27^KIP1^, CDKN1A/p21), cell death (e.g., FasL, Trail, Bim), and metabolism [32–34]. In addition, forkhead box O (FOXO) factors play important anti-tumoral activities by interfering with senescence induced by oncogenes, angiogenesis, resistance to oxidative stress, and the control of cell invasion [35]. In prostate cancer, astrocyte-elevated gene-1 (AEG1) is often over-expressed and plays a role in cell invasion. AEG1 knock-down reduces cell viability and invasiveness and increases FOXO3 expression and its nuclear localization. FOXO3 expression is decreased in invasive urothelial cancer and correlates with patient survival [36]. Aberrant activation of Ras triggers senescence through a negative feedback loop that suppresses Ras and PI3K signaling, leading to activation of FOXO1 and 3 [37]. FOXO factors play important roles in tumor progression and metastasis [38]. FOXOs are targeted and inactivated by the PI3K-AKT axis [39]. Nevertheless, the underlying role of FOXOs is not fully understood in cancer.

In the present study, we show that cisplatin (CDDP) induced ubiquitylation of AKT and FOXO3 plays an important role in this process through MUL1 regulation.

## RESULTS

### Cisplatin induces thyroid cancer cell death through AKT downregulation

**Figure 1:**
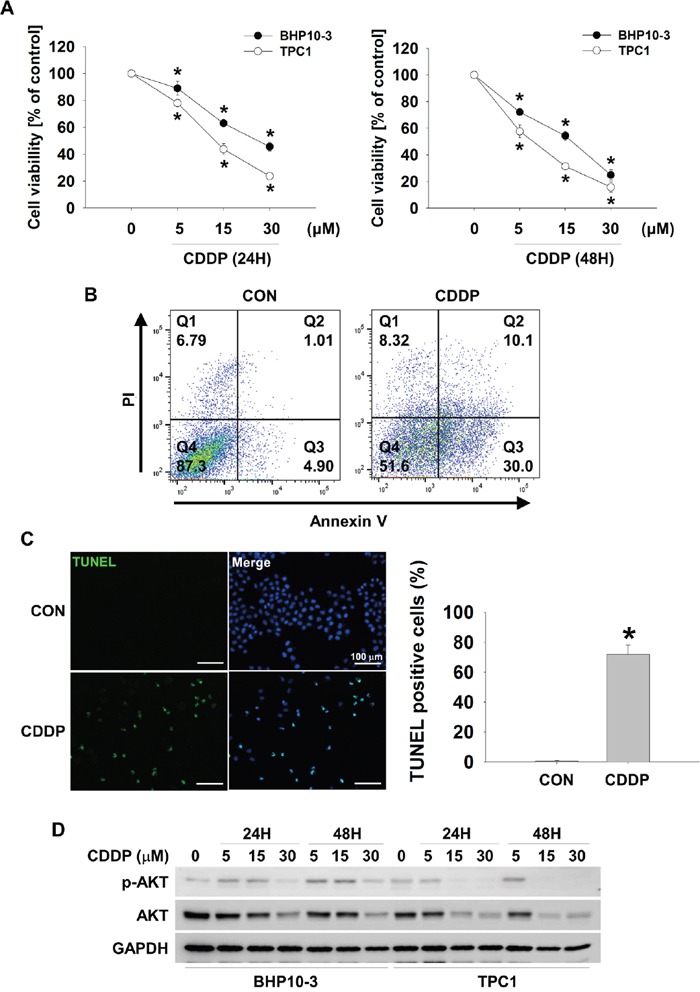
CDDP causes downregulation of p-AKT and AKT and increases cell cytotoxicity in thyroid cancer cells **(A** and **B)** CDDP reduces thyroid cancer cells viability. BHP10-3 and TPC1 cells were treated with CDDP at the indicated concentrations in the absence of serum for 24 hours (*left*) or 48 hours (*right*). Cell viability was evaluated by MTT assay (A, n = 6), FACS analysis (B) and TUNEL assay **(C)**. Data are mean ± SD. Asterisks indicate statistically significant differences (*P < 0.05*). **(D)** p-AKT and AKT decrease in response to CDDP. BHP10-3 and TPC1 cells were treated with indicated concentrations of CDDP for 24 hours or 48 hours in the absence of serum and the reduction in p-AKT and AKT was assessed by Western blot.

To determine whether CDDP could induce thyroid cancer cell death, we treated CDDP to BHP10-3 and TPC1 thyroid cancer cells in a time dependent or dose dependent fashion. Both thyroid cancer cells underwent death by CDDP treatment (Figure 1A). TPC1 cells died more rapidly than BHP10-3 cells by CDDP treatment. CDDP-induced thyroid cancer cell death was confirmed by FACS analysis and TUNEL assay (Figure 1B and 1C). AKT is a well-known oncogenic protein which protects cancer cells from extracellular stress or controls the proliferation of cancer cells [2]. For these reasons, we evaluated whether CDDP could induce downregulation of AKT in thyroid cancer cells. CDDP was treated in a dose or time dependent fashion and p-AKT and total AKT was determined by Western blot assay. CDDP induced downregulation both p-AKT and total AKT in BHP10-3 and TPC1 cells. However, CDDP-induced AKT reduction was less pronounced in BHP10-3 than TPC1 suggesting relative resistance (Figure 1D). In TPC1 cells, AKT downregulation was induced from 15 μM of CDDP. These data indicated that CDDP-induced thyroid cancer cell death involves AKT downregulation.

### CDDP leads to ubiquitylation of AKT

**Figure 2:**
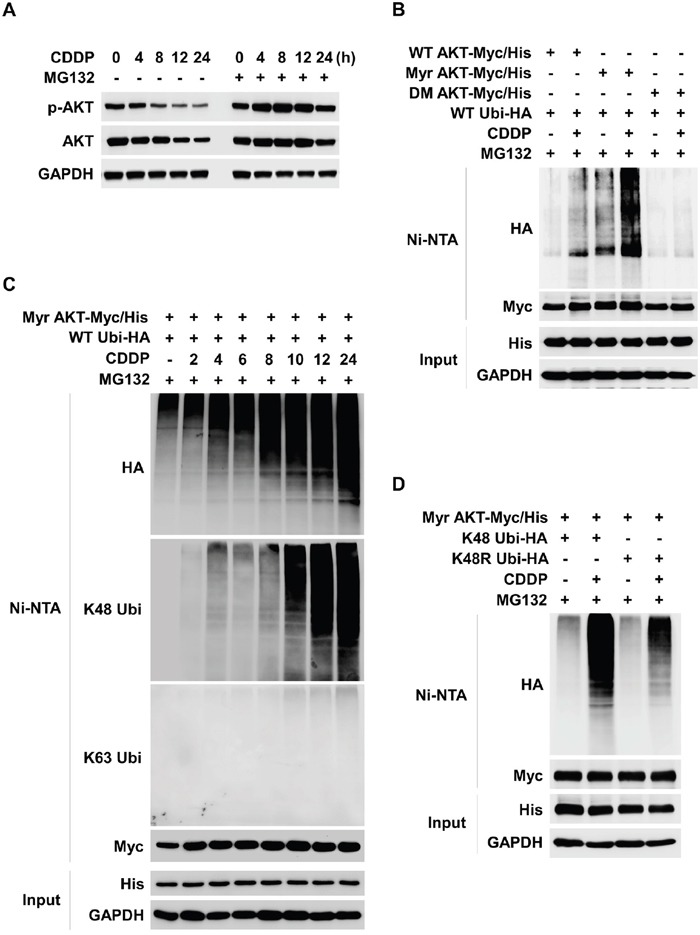
CDDP induces K48-linked ubiquitylation and degradation of AKT **(A)** CDDP induces ubiquitin proteasome system (UPS) of AKT. TPC1 cells were treated with CDDP for each indicated times. MG132 (1 μM) was pre-treated for 1 hour before the CDDP treatment. p-AKT and AKT expressions were evaluated by Western blot. **(B)** TPC1 cells were transfected with the wild-type AKT (WT AKT/Myc-His), constitutively active myristoylated AKT (Myr-AKT/Myc-His), or inactive AKT (DM AKT/Myc-His) plasmid together with the hemagglutinin-tagged ubiquitin (Ubi-HA) plasmid. After 24 hours, CDDP was treated for 24 hours with MG132 (1 μM) to TPC1 cells in the absence of serum. Ubiquitylated AKT was detected by Ni-NTA His pull-down and Western blot. **(C)** TPC1 cells were transfected with the Myr AKT/Myc-His plasmid together with the Ubi-HA plasmid. 24 hours after transfection, TPC1 cells were treated with CDDP for the indicated times in the absence of serum. Ubiquitylated AKT was detected by Ni-NTA His pull-down and Western blot. **(D)** TPC1 cells were transfected with Myr AKT/Myc-His plasmid together with the lysine mutant HA-tagged Ubi plasmid (K48 Ubi-HA, all lysine residues except Lys-48 replaced with arginine; K48R Ubi-HA, Lys-48R replaced with arginine). 24 hours after transfection, TPC1 cells were treated with CDDP for 24 hours in the absence of serum.

CDDP treatment induced downregulation of both p-AKT and total AKT and led to death of thyroid cancer cells (Figure 1). Therefore, we determined whether CDDP could induce post transcriptional modification (PTM) such as UPS. CDDP decreased levels of p-AKT and total AKT in a time dependent manner however, these decrements were inhibited by a proteasome inhibitor, MG132 (Figure 2A). To clarify CDDP-induced ubiquitylation of AKT, we transfected TPC1 with wild type (WT), active (myristorylated, Myr), or inactive (T308/S473 double mutant, DM) AKT series and investigated AKT ubiquitylation by Ni-NTA His pull-down assay. As a result, CDDP induced ubiquitylation of AKT. Specifically, active form of AKT was strongly ubiquitylated by CDDP however, the inactive form was not ubiquitylated (Figure 2B). CDDP induced AKT ubiquitylation in a time dependent manner (Figure 2C). CDDP-mediated AKT ubiquitylation was identified as K48-linked ubiquitylation (Figure 2C, anti-K48 blot). To confirm the CDDP-mediated K48-linked ubiquitylation of AKT, we used K48 mutant ubiquitin plasmid (K48R). CDDP strongly induced K48-linked ubiquitylation of AKT however, K48R ubiquitin did not lead to AKT ubiquitylation (Figure 2D). Taken together, these data indicated that CDDP induced downregulation of AKT through K48-linked UPS.

### MUL1 ubiquitylates AKT by CDDP

**Figure 3:**
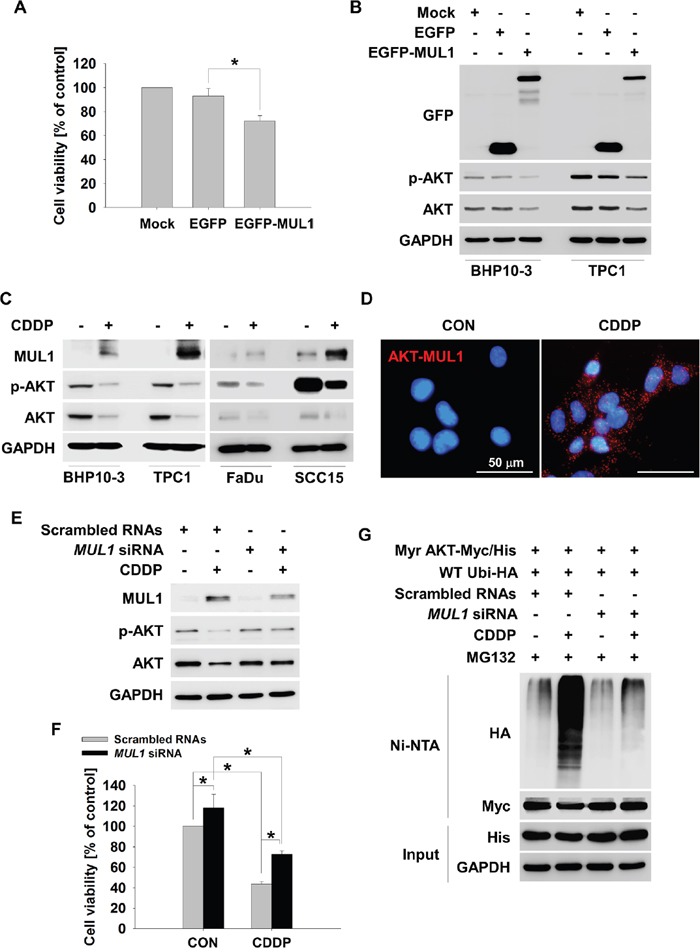
MUL1 is an E3 ligase responsible for CDDP-induced AKT ubiquitylation **(A)** MUL1 induces cell cytotoxicity. TPC1 cells were transfected with Mock, EGFP or EGFP-MUL1 plasmid, respectively. At 24 hours after transfection, TPC1 cells were seeded in a 48-well plate and incubated for 24 hours. Cell viability was measured by the MTT assay (n = 6). Data are mean ± SD. Asterisks indicate statistically significant differences (*P < 0.05*). **(B)** MUL1 overexpression reduces p-AKT and AKT. BHP10-3 and TPC1 cells were each transfected with Mock, EGFP, and EGFP-MUL1 plasmid respectively, for 24 hours followed by cell lysis for Western blot. **(C)** CDDP induces MUL1 expression. Thyroid cancer or HNC cells (FaDu and SCC15) were treated with CDDP for 24 hours in the absence of serum, and each protein levels were determined by Western blot. **(D)** CDDP increased the level of the interaction between MUL1 and AKT. TPC1 cells were seeded at coverslip and then, CDDP was treated for 12 hours. The interaction between AKT and MUL1 was determined by PLA assay. Fluorescence images were observed by confocal microscopy. Scale bar represents 50 μm. **(E)** CDDP-induced AKT downregulation is associated with MUL1. TPC1 cells were transfected with scrambled RNAs or *MUL1* siRNA and 24 hours later, CDDP was treated for 24 hours in the absence for serum and each protein levels were determined by Western blot. **(F)** MUL1 knock-down rescues CDDP-induced cytotoxicity. Scrambled RNAs or *MUL1* siRNA transfected TPC1 cells were seeded in a 48-well plate and then, CDDP was treated for 24 hours in the absence of serum. Cell viability was measured by MTT assay (n = 6). Data are mean ± SD. Asterisks indicate statistically significant differences (*P < 0.05*). **(G)** MUL1 knock-down prevents CDDP-induced AKT ubiquitylation. TPC1 cells were transfected with scrambled RNAs or *MUL1* siRNA and 24 hours later, Myr-AKT/Myc-His and Ubi-HA plasmid were transfected for 12 hours. CDDP was treated for 24 hours with MG132 (1 μM) in the absence of serum. The cell lysates were subjected to denatured Ni-NTA His pull-down and Western blot.

MUL1 has been previously identified as a E3 ligase against AKT [30]. We reported that MUL1 regulation was associated with head and neck cancer (HNC) development through reduction of AKT [31]. Following these reports, we hypothesized that MUL1 suppression may also contribute to development of thyroid cancer, thus we checked the relationship between CDDP-induced ubiquitylation of AKT and MUL1 induction. To determine whether MUL1 is associated with thyroid cancer cell survival, we transfected EGFP-MUL1 in TPC1 cells and measured cell viability. MUL1 overexpressed TPC1 cells presented decreased cell viability compared with control or EGFP transfected cells (Figure 3A). MUL1 overexpression reduced both p-AKT and total AKT levels in BHP10-1 and TPC1 thyroid cancer cells (Figure 3B). These data indicated that MUL1 acts as a negative regulator in thyroid cancer cells. Regarding that CDDP induced K48-linked ubiquitylation of AKT (Figure 2), we checked the MUL1 expression level in response to CDDP treatment in thyroid cancer cells. CDDP treatment increased MUL1 levels in both BHP10-1 and TPC1 cells followed by AKT reduction (Figure 3C). The interaction between AKT and MUL1 was increased by CDDP treatment in proximity ligation assay (PLA) (Figure 3D). These results indicated that CDDP-induced AKT downregulation was closely associated with MUL1. Therefore, we examined the role of MUL1 in CDDP-mediated AKT ubiquitylation. CDDP-induced AKT downregulation was prevented by *MUL1* siRNA treatment (Figure 3E). CDDP-induced cytotoxicity was inhibited in MUL1 knock-downed cells compared with control (Figure 3F). AKT ubiquitylation in response to CDDP was accumulated however, the AKT ubiquitylation was prevented in *MUL1* siRNA treatment condition (Figure 3G). Reflecting these results, it is suggested that CDDP induced MUL1 induction which in turn results in AKT ubiquitylation, thereby leading to cell death.

### FOXO3 plays an important role in CDDP-induced MUL1 expression

**Figure 4:**
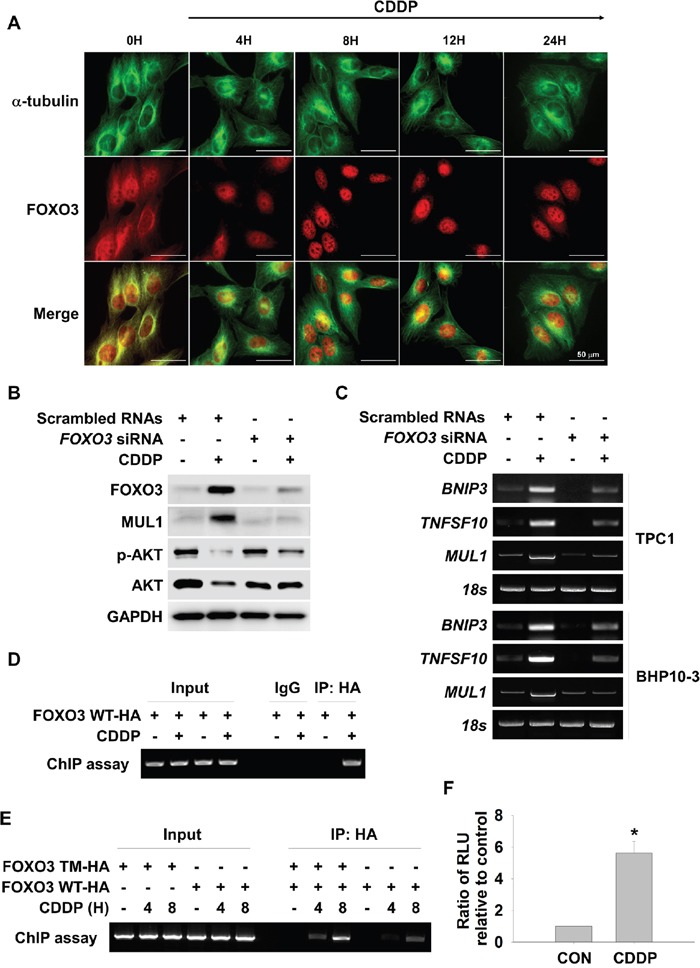
FOXO3 mediates CDDP-induced MUL1 expression **(A)** CDDP causes FOXO3 translocation into the nucleus. TPC1 cells were cultured on coverslips and treated with CDDP in the absence of serum for the indicated times. Immunofluorescent staining was then performed using the anti-FOXO3 (*red*), anti-Tubulin (*green*) antibody and DAPI (*blue*). Scale bar represents 50 μm. Tubulin and DAPI were also each applied to visualize the cytoplasm and nuclei, respectively. **(B** and **C)** FOXO3 knock-down inhibits CDDP-mediated MUL1 induction. TPC1 and BHP10-3 cells were transfected with scrambled RNAs or *FOXO3* siRNA. At 24 hours after siRNA transfection, CDDP was treated for 12 or 24 hours in the absence of serum for Western blot and *MUL1* RT-PCR. *BINP3* or *TNFSF10* was used as positive control against FOXO3. **(D)** CDDP induces increased activity of FOXO3 DNA binding. FOXO3 plasmids were transfected into TPC1 cells and then, cells were cultured with or without CDDP for 24 hours. FOXO3 DNA binding activity was determined with ChIP assay. **(E)** FOXO3 triple mutant (FOXO3 TM) or FOXO3 wild type (FOXO3 WT) plasmids were transfected to TPC1 and then, CDDP was treated for 24 hours. **(F)** CDDP induces increased level of FOXO3 reporter activity. The TPC1 cells were cotransfected with FOXO3 reporter plasmids (pGL4-FOXO3/Luc) and FOXO3. After 24 hours of incubation, cells were lysed and the reporter luciferase activities driven by the FOXO3 were determined.

CDDP induced K48-linked ubiquitylation of AKT through MUL1 dependent manner (Figure 3). MUL1 was considered to be a tumor suppressor protein in thyroid cancer, therefore further investigation was conducted in the interest of identifying regulators of MUL1. Using the TRANSFAC^®^ database, we analyzed putative regulator binding sites in the human MUL1 gene promoter about 2 kb from the transcription initiating site. We found FOXO3 as a putative transcriptional regulator against MUL1, thus we analyzed relationship between FOXO3 and MUL1 under CDDP treatment. FOXO1 and FOXO4 levels were mildly increased against CDDP, however, FOXO3 was strongly accumulated in CDDP-treated cells (Supplementary Figure 1). Therefore, we tested whether CDDP could induce translocation of FOXO3, so CDDP was treated to TPC1 cells in a time dependent manner and FOXO3 location was observed by immnunocytochemistry. FOXO3 was translocated from the cytosol to the nucleus in a time dependent fashion in response to CDDP (Figure 4A and Supplementary Figure 2). More specifically, FOXO3 was observed in the nucleus from 4 hours of CDDP treatment. CDDP induced accumulation of MUL1 followed by FOXO3 increment, however, FOXO3 knock-down did not induce MUL1 induction and also AKT downregulation (Figure 4B). To confirm the relationship between CDDP-induced FOXO3 increment and MUL1 induction, we checked the *MUL1* gene expression level. While *MUL1* gene was expressed by CDDP treatment, FOXO3 knock-down inhibited CDDP-induced *MUL1* expression (Figure 4C). To confirm the relationship between FOXO3 and MUL1, we determined FOXO3 activity through chromatin immunoprecipitation assay (ChIP). We analyzed putative FOXO3 binding region in the 5’ upstream of the MUL1 gene spanning approximately 1.7 kb from the transcription start site (Supplementary Figure 3). CDDP induced increased binding of FOXO3 in the 5’ upstream region of MUL1 gene (Figure 4D). FOXO3 phosphorylation sites mutant (T32A, S253A and S315A) against AKT (FOXO3 triple mutant, FOXO3 TM) was strongly bound at *MUL1* promoter region by CDDP treatment (Figure 4E). To clarify FOXO3-mediated MUL1 regulation, we generated FOXO3 response element luciferase system which contained 1.7 kb from the transcription start site of *MUL1* gene and checked CDDP-induced FOXO3 transcriptional activity. The FOXO3-mediated reporter activity was increased by CDDP treatment (Figure 4F). For these reasons, MUL1-AKT interaction was not observed in *FOXO3* siRNA treated cells by proximity ligation assay (PLA) (Supplementary Figure 4). These data indicated that FOXO3 acts as a regulator of MUL1 in response to CDDP.

### FOXO3-MUL1 axis regulates ubiquitylation of AKT through ROS dependent manner

**Figure 5:**
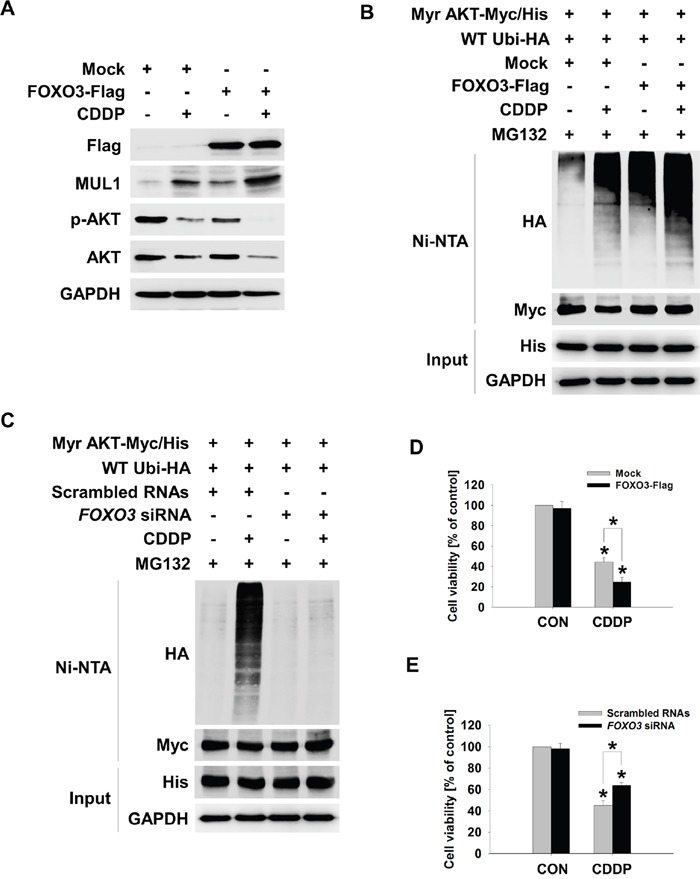
FOXO3 mediates CDDP-induced MUL1 expression **(A** and **B)** FOXO3 promotes CDDP-induced MUL1 expression and AKT ubiquitylation. (A) TPC1 cells were transfected with Mock or FOXO3-Flag plasmid and then, CDDP was treated for 24 hours in the absence of serum. The cells were subjected to Western blot assay with the indicated antibodies. (B) TPC1 cells were transfected with Mock or FOXO3-Flag together with the Myr-AKT/Myc-His and Ubi-HA plasmid. At 24 hours later, CDDP was treated for 24 hours with MG132 (1 μM) in the absence of serum. The cell lysates were subjected to denatured Ni-NTA His pull-down and Western blot. **(C)** FOXO3 knock-down inhibits CDDP-mediated AKT ubiquitylation. TPC1 cells were transfected with scrambled RNAs or *FOXO3* siRNA and 24 hours later, Myr-AKT/Myc-His and Ubi-HA plasmid were transfected for 12 hours. CDDP was treated for 24 hours with MG132 (1 μM) in the absence of serum. The cell lysates were subjected to denatured Ni-NTA His pull-down and Western blot. **(D)** Mock or FOXO3-Flag plasmid transfected TPC1 cells were seeded in a 48-well plate and then, CDDP was treated for 24 hours in the absence of serum. Cell viability was measured by MTT assay (n = 6). Data are mean ± SD. Asterisks indicate statistically significant differences (*P < 0.05*). **(E)** Scrambled RNAs or *FOXO3* siRNA transfected TPC1 cells were seeded in a 48-well plate and then, CDDP was treated for 24 hours in the absence of serum. Cell viability was measured by MTT assay (n = 6). Data are mean ± SD. Asterisks indicate statistically significant differences (*P < 0.05*).

**Figure 6:**
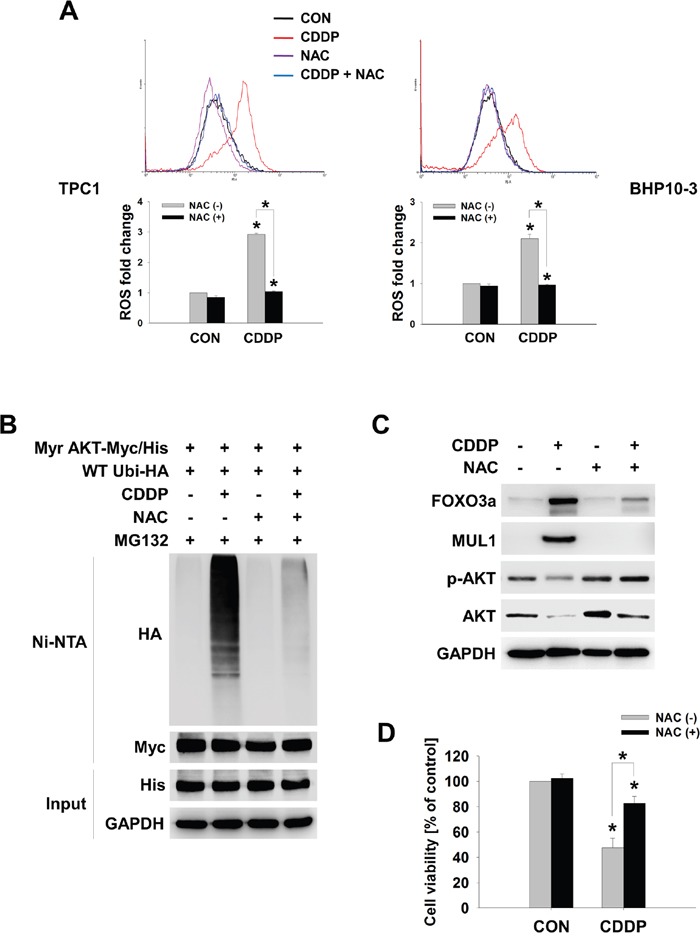
CDDP-induced cellular ROS controls the FOXO3-MUL1-AKT axis **(A)** CDDP-induced ROS increment is inhibited by NAC. BHP10-3 and TPC1 cells were pre-treated with NAC (10 mM) for 1 hour and then, CDDP was treated for further 24 hours. Intracellular ROS levels were determined by FACS analysis (n=3). Data are mean ± SD. Asterisks indicate statistically significant differences (*P < 0.05*). **(B)** NAC inhibits CDDP-induced AKT ubiquitylation. Myr-AKT/Myc-His together with Ubi-HA transfected TPC1 cells were pre-treated with NAC (10 mM) for 1 hour and treated with or without CDDP in the absence of serum for 24 hours. Ubiquitylated AKT was detected by Ni-NTA His pull-down and Western blot. **(C)** NAC inhibits CDDP-induced MUL1 expression and AKT degradation by CDDP. TPC1 cells were pre-treated with NAC for 1 hour and then, CDDP was treated for 24 hours in the absence of serum. Each protein levels were determined by Western blot. **(D)** NAC inhibits CDDP-mediated cytotoxicity. TPC1 cells were seeded at 48-well plate and NAC was pre-treated for 1 hour. After then, NTS was treated further 24 hours with or without NAC. Cells viability were measured by MTT assay (n = 6). Data are mean ± SD. Asterisks indicate statistically significant differences (*P < 0.05*).

**Figure 7:**
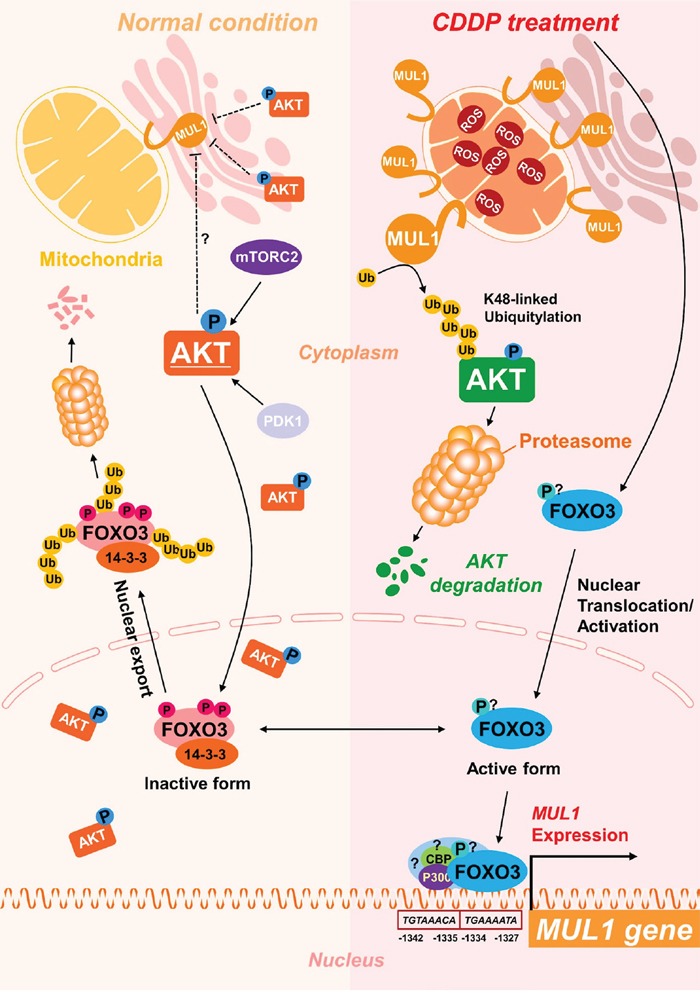
Scheme of FOXO3-MUL1-AKT axis response to CDDP FOXO3 is inactivated in normal condition through phosphorylation by AKT or ubiquitylation, however, CDDP induces accumulation of MUL1 and leads to degradation of AKT through K48-linked ubiquitylation. Furthermore, CDDP-induced FOXO3 activation controls induction of MUL1.

FOXO3 was involved in CDDP-mediated MUL1 induction and AKT reduction, thus we tested whether FOXO3-MUL1 axis could contribute to ubiquitylation of AKT. We transfected TCP-1 cells with FOXO3 (FOXO3-Flag) and treated with CDDP. While CDDP treatment increased MUL1 level and decreased AKT, FOXO3 overexpression induced MUL1 expression in the absence of CDDP (Figure 5A). CDDP-mediated MUL1 induction or AKT decrement was enhanced by FOXO3 overexpression. CDDP induced accumulation of AKT ubiquitylation, however ubiquitylation of AKT was enhanced by FOXO3 (Figure 5B). In contrast with FOXO3 overexpression, CDDP-induced ubiquitylation of AKT was prevented by *FOXO3* siRNA treatment (Figure 5C). For these reasons, FOXO3 overexpression showed synergistic effect together with CDDP treatment (Figure 5D). FOXO3 knock-downed cells were resistant from CDDP treatment (Figure 5E). These data indicated that FOXO3 has a pivotal role in ubiquitylation of AKT through MUL1 regulation. Previously, we reported that intracellular ROS plays an important role in MUL1-mediated AKT ubiquitylation in HNC [30]. CDDP could increase ROS levels [40], so we determined whether the FOXO3-MUL1-AKT axis was involved in CDDP-mediated intracellular ROS production. CDDP increased intracellular ROS levels in both TPC1 and BHP10-3 thyroid cancer cells (Figure 6A). CDDP-induced ubiquitylation of AKT was inhibited by a ROS scavenger, N-acetylcysteine (NAC) (Figure 6B). AKT ubiquitylation induced by CDDP was prevented in NAC pre-treated cells, thus we further checked the relationship between FOXO3 and MUL1 under these conditions. CDDP induced FOXO3 and MUL1 while p-AKT and total AKT were decreased, however FOXO3 and MUL1 increases were inhibited by NAC (Figure 6C). p-AKT and total AKT reductions were also prevented, therefore CDDP could not induce ubiquitylation of AKT in NAC pre-treated cells (Figure 6B). Followed by these results, CDDP could not induce cytotoxicity in NAC treated cells compared with controls (Figure 6D). Based on these data, we could suggest that CDDP induced K48-linked ubiquitylation of AKT through FOXO3-MUL1 axis and ROS is important in the CDDP-mediated FOXO3-MUL1-AKT signal pathway. From these findings, we suggest that FOXO3-MUL1 axis could be a therapeutic target for novel cancer strategy for the treatment of HNC with CDDP (Figure 7).

## DISCUSSION

In the present study, we show that AKT was ubiquitylated by CDDP and FOXO3 plays a pivotal role during this process, through MUL1 regulation. CDDP induced K48-linked ubiquitylation of AKT in a time dependent manner. CDDP-mediated AKT ubiquitylation was suppressed by MUL1 knock-down, so we then searched for regulators of MUL1 by bioinformatic methods and determined the role of FOXO3 as a positive regulator of MUL1 induction. CDDP induced nuclear accumulation of FOXO3 and MUL1 expression was dependent on FOXO3. Ultimately, CDDP-mediated FOXO3-MUL1-AKT axis was controlled by the intracellular ROS system.

Protein degradation by UPS is used to control many cellular processes [25]. Several reports have described UPS-mediated AKT proteolytic degradation in different types of cells [41]. Of note, MUL1 induced p-AKT ubiquitylation in a specific manner [30]. The results in the present study also showed that CDDP induced ubiquitylation of active AKT (Figure 2B) and these events were inhibited by MUL1 knock-down (Figure 3G). From the previous reports of our study, MUL1 plays a pivotal role in HNC progression through its suppression [31]. AKT is also overexpressed and hyperactivated in thyroid cancer [42]. In thyroid cancer cells, a chemopreventive non-steroidal anti-inflammatory drug, sulindac sulfide, blocks the PI3K-AKT pathway and leads to the activation of FOXO3, which increases the expression of Bim, GADD45A and p27^KIP1^ to promote cell cycle arrest and apoptosis [43]. From the results of the present study, MUL1 was suppressed endogenously in thyroid cancer cells (Figure 3), thus MUL1 may act as a thyroid cancer suppressor protein through regulation of AKT activity.

AKT controls the functions of many proteins through phosphorylation. One of the target proteins of AKT is FOXO3 [39]. The expression and activity of FOXO factors are strongly controlled by PTM such as phosphorylation, acetylation, methylation and ubiquitylation [44]. A major mechanism of FOXO3 regulation consists of phosphorylation by AKT on three residues, T32, S253, and S315 by growth factor stimulation [39], leading to FOXO3 inactivation. For these reasons, FOXO factors are well-established mediators of cancer cell death induced by chemotherapeutic agents [45]. In breast cancer cells treated with 5-fluorouracil, there is an accumulation of human antigen R (HuR), an RNA-binding protein which binds to and stabilizes FOXO1 mRNA to promote apoptosis [46]. Paclitaxel, which is also used for the treatment of breast cancer, was shown to stimulate apoptosis via FOXO3-induced Bim expression [47]. Similarly, FOXO3 is associated with the apoptotic effect of imatinib in leukemia cells via Bim induction [48]. Other anti-cancer drugs (e.g. Trastuzumab, Lapatinib and Tamoxifen) activate FOXOs to mediate apoptosis in cancer cells [49]. ONC201/TIC10 is a dual inhibitor of AKT and ERK that targets chemotherapy-resistant colorectal cancer stem cells via induction of TRAIL expression by FOXO3 [50]. CDDP induced colon, lung, and oral cancer cell death through FOXO3 mediation [51–55]. In the present study, we also showed that CDDP induced thyroid cancer cells death via FOXO3 increment. Furthermore, our data showed that CDDP led to downregulation of AKT and it was associated with FOXO3-mediated AKT ubiquitylation (Figure 5). FOXO3 was determined as a putative regulator of MUL1, therefore FOXO3 and MUL1 were suppressed in thyroid cancer cells (Figure 4B). The suppression of FOXO3-MUL1 maybe lead to overexpression or hyperactivation of AKT in thyroid cancer [42]. Thus, FOXO3 could be a good therapeutic target for thyroid cancer therapy through induction of the MUL1-AKT axis.

CDDP is a first-line chemotherapeutic agent in the therapy of various human cancers. The antitumor mechanisms of CDDP include failure to repair damaged DNA, building bifunctional DNA crosslinks, interference with replication of DNA, and induction of cell apoptosis/necrosis [56–58]. Despite its significant effect on cancers, CDDP still has some disadvantages such as drug resistance. CDDP could not be used in the clinic because thyroid cancer presented drug resistance against CDDP. However, on the basis of the results of this study, we suggest that CDDP resistance could be overcome if the FOXO3-MUL1 axis could be regulated. Although more detailed experiments are necessary to elucidate the underlying expression profiling between FOXO3 and MUL1 in tissues of thyroid cancer patients, we could speculate that while p-AKT is increased, FOXO3 and MUL1 is decreased.

In the present study, we showed that CDDP-mediated FOXO3 nuclear accumulation increased MUL1 level and led to K48-linked ubiquitylation of active AKT. Therefore, we suggest that FOXO3-MUL1 axis could be a good therapeutic target for cancer therapy using the CDDP.

## MATERIALS AND METHODS

### Reagents and antibodies

Chemicals were obtained from the following sources: *cis*-Diammineplatinum (II) dichloride (CDDP), N-acetylcysteine (NAC) and MG132 were purchased from Sigma-Aldrich (St. Louis, MO, USA). Antibodies were obtained from the following sources: anti-AKT (#9272 for western blot), anti-AKT (#2920 for proximity ligation assay, PLA), anti-p-AKT (Ser473), anti-Myc, anti-His, anti-FOXO1, anti-FOXO3, anti-FOXO4, anti-GAPDH, anti-Normal Rabbit IgG, horseradish peroxidase (HRP)-conjugated anti-mouse IgG and anti-rabbit IgG (Cell signaling Technology, Beverly, MA, USA), anti-K48 Ubiquitin, anti-K63 Ubiquitin, anti-α-Tubulin (Millipore, Temecula, CA, USA), anti-HA for western blot, anti-GFP (Santa Cruz Biotechnology, Santa Cruz, CA, USA), anti-MUL1, anti-Lamin A and anti-HA for ChIP assay (abcam, Cambridge, MA, USA), anti-hemagglutinin (HA), anti-GFP (Santa Cruz, Biotechnology, Santa Cruz, CA, USA), anti-Flag (Sigma-Aldrich), Alexa Flour 488-conjugated goat anti-Mouse and Alexa Flour 546-conjugated goat anti-Rabbit (Invitrogen, Carlsbad, CA, USA)

### Cell culture

Thyroid cancer (TPC1 and BHP10-3) cells and head and neck cancer (HNC; FaDu and SCC15) cells were purchased from the American Type Culture Collection (ATCC; Manassas, VA, USA). TPC1 and BHP10-3 cells were grown in Roswell Park Memorial Institute 1640 (RPMI 1640; GIBCO, Carlsbad, CA, USA), while FaDu cells were maintained in Minimum Essential Medium (MEM; GIBCO), while SCC-15 cells were grown in Dulbecco's modified Eagle's medium/Ham's nutrient mixture F-12 (DMEM/F12; GIBCO), supplemented with 10% fetal bovine serum (FBS; GIBCO) and 100 U/ml penicillin-streptomycin (GIBCO) at 37°C with 5% CO_2_ under humidified conditions.

### Plasmid transfection

The entire coding regions of MUL1 were generated by PCR from cDNA synthesized from Human Universal QUICK-CloneTM II (Clontech Laboratories, Inc). Human MUL1 cDNA was cloned into pEGFP-N1 (Clontech). The sequences of primers used for PCR are as follows: human MUL1, 5’-CTA GCT AGC TAG ATG GAG AGC GGA GGG CGG CCC T-3’ and 5’-CCG CTC GAG CGG GCT GTT GTA CAC GGG TAT CAC C-3’. FOXO3-Flag, FOXO3 WT-HA and FOXO3 triple (T32A, S253A, S315A) mutant-HA (FOXO3 TM-HA) plasmid [39] was obtained from Addgene (Cambridge, MA, USA). The pcDNA3.1-wild-type AKT-Myc/His (WT AKT-Myc/His), pcDNA3.1-myristoylated AKT-Myc/His (Myr AKT-Myc/His), pcDNA3.1-T308A/S473A double mutant AKT-Myc/His (DM AKT/Myc-His), wild-type ubiquitin-HA (WT Ubi-HA), mutant K48 ubiquitin-HA (K48 Ubi-HA) and mutant K48R ubiquitin-HA (K48R Ubi-HA) plasmids were used as described previously [30]. To generate the MUL1 luciferase promoter constructs (-1566 to +100), the following 5’ upstream regions of MUL1 gene were amplified from Human Genomic DNA (TaKaRa, Madison, WI, USA) and inserted into pGL4.23 [*luc2*/minP] vector (Promega). The sequences of primers used for PCR are as follows: 5’-GCT AGC ATG AAG TTG CAC TGA GCC GAG ATC-3’ and 5’-CCC TCG AGG GGG GCC TTC TGC CGG TAC ACG G-3’. Transient transfections were performed with Lipofectamine 2000 (Invitrogen), according to the manufacturer's instructions. Briefly, when cells reached 70% confluence in a 10-cm diameter culture dish, the culture medium was replaced with opti-MEM (Invitrogen), and each plasmids (1μg) were transfected. After 6 hours, the culture medium was replaced with RPMI 1640 containing 10% FBS, and TPC1 cells were incubated for 48 hours. pcDNA3.1-Myc/His was used as a mock transfection control.

### Cell viability assay

Human thyroid cancer (TPC1 and BHP10-3) cells were seeded at 150 cells/mm^2^ for 24 hours on 48-well plates and then grown in medium containing FBS. On the following day, cells were washed with PBS and treated with CDDP in the absence of serum. Cell viability was measured with a Thiazolyl Blue Tetrazolium Bromide (MTT) assay (Sigma-Aldrich) according to the manufacturer's instructions.

### Annexin V and propidium iodide (PI) analysis

Quantitative apoptotic cell death by CDDP was detected using the Annexin V-propidium iodide (PI) apoptosis detection kit I (BD Biosciences, Bedford, MA, USA) according to the manufacturer's instructions. TPC1 cells were treated with CDDP for 16 hours. The cells were harvested, washed with cold phosphate-buffered saline (PBS), and stained with Annexin V-fluorescein isothiocyanate and PI at room temperature for 15 min in the dark. The early and late apoptosis were quantified according to the manufacturer's instructions. Apoptosis was detected using a FACS Aria system (BD Biosciences), with the excitation and emission settings of 488 and 530 nm, respectively.

### Western blot analysis

Cells were lysed with la radio-immunoprecipotation assay (RIPA) lysis buffer (20 mM Tris-HCl, pH 7.5, 150 mM NaCl, 1 mM EDTA, 1 mM EGTA, 1% Triton X-100, 0.1% SDS, 1% Nonidet P-40, 2.5 mM sodium pyrophosphate, 1 mM β-glycerophosphate, 1 mM Na_3_VO_4_, 2 mM *p*-nitrophenyl phosphate, and a protease inhibitor cocktail) on ice for 30 min. Following centrifugation at 14,000 ×g for 20 min at 4°C, the proteins in supernatants were separated by sodium dodecyl sulfate-polyacrylamide gel electrophoresis (SDS-PAGE), and transferred to a polyvinylidene difluoride (PVDF) membrane (Millipore). Membranes were blocked with 5% skim milk for 1 h at room temperature and incubated overnight with primary antibody (1:1,000) at 4°C. After washing with 0.1% Tween-20 in Tris-buffered saline (TBS-T), membranes were then incubated with HRP-conjugated secondary antibody (1: 5,000). Proteins were visualized using ECL reagents (GE Healthcare Life Sciences, New York, NY, USA) and detected with an LAS-4000 imaging system (Fujifilm, Tokyo, Japan). Image densities were quantified with ImageJ analysis software.

### RNA interference analysis

A total of 300 pmols of scrambled RNAs or siRNAs were transfected into TPC1 cells at 70% confluency in a 10-cm diameter culture dish using Lipofectamine® RNAiMAX Transfection Reagent (Thermo Fisher, Pittsburgh, PA, USA), according to the manufacturer's instructions. After 24 hours, transfected TPC1 cells were treated with CDDP. Duplexes of siRNA targeting *MUL1* and *FOXO3* were synthesized by BIONEER (Daejeon, Korea). The siRNA sequences are as follows: human *MUL1*, 5’-GUA CAA CAG CUA AUA GUU U-3’ and 5’-A AAC UAU UAG CUG UUG UAC-3’; human *FOXO3*, 5’-CAA CCU GUC ACU GCA UAG U-3’ and 5’-A CUA UGC AGU GAC AGG UUG-3’.

### RT-PCR

Expression of *MUL1* and *18s* was estimated by RT-PCR. Total RNAs in TPC1 and BHP10-3 cells were isolated using TRIzol reagent (Thermo Fisher) and cDNAs were synthesized with 1 μg of total RNAs and ReverTra Ace^®^ qPCR RT Master Mix (TOYOBO, Osaka, Japan), according to the manufacturer's instructions. PCR primer sequences were as follows: human *MUL1*, 5’-CAC AAG ATG GTG TGG AAT CG-3’ and 5’-TCA GCA TCT CCT CGG TCT CT-3’; human *TNFSF10*, 5’-GAC CCC AAT GAC GAA GAG AG-3’ and 5’-TCC TTG ATG ATT CCC AGG AG-3’; human *bNIP3*, 5’-CTG GAC GGA GTA GCT CCA AG-3’ and 5’-TCT TCA TGA CTC TCG TGT TC-3’; human *18s*, 5′-CAC GGA CAG GAT TGA CAG AT-3′ and 5′-CGA ATG GGG TTC AAC GGG TT-3′. PCR products were separated by 1% agarose gel, stained with GelRed Nucleic acid gel stain (Biotium, Fremont, CA, USA), and visualized using a ImageQuant™ LAS 4000 (FujiFilm).

### TdT-mediated dUTP nick end labeling (TUNEL) assay

Analysis of apoptosis was performed using TUNEL. TPC1 cells were seeded on glass cover slips (Thermo Fisher), cultured overnight in a 12-well plate, and treated with CDDP for 16 hours. TPC1 cells were fixed with 4% paraformaldehyde in PBS for 10 min, washed three times with PBS, permeabilized with 0.1% Triton X-100 in PBS for 2 min on ice, and washed twice in PBS. Staining was performed by incubating cells for 1 hour at 37°C in 50 μl of TUNEL reaction mixture in *In Situ* Cell Death Detection Kit, Fluorescein (Sigma-Aldrich), according to the manufacturers’ protocol. After nuclei were stained using Hoechest (Thermo Fisher), cells were observed with a confocal laser microscope (Nikon, Tokyo, Japan).

### *In situ* proximity ligation (PLA) assay

At 70% confluence, TPC1 cells were seeded on glass cover slips (Thermo Fisher), cultured overnight in a 12-well plate, and treated with CDDP for 12 hours. Thereafter, TPC1 cells were fixed with 4% paraformaldehyde in PBS for 10 min, permeabilized with 0.1% Triton X-100 in PBS for 5 min, and incubated with 5% BSA for 1 hour at room temperature. Cells were incubated with rabbit anti-MUL1 (1:100) and mouse anti-AKT (1:100) antibodies at 4°C overnight. Cells were incubated with anti-rabbit PLUS and anti-mouse MINUS PLA probes in Duolink *in situ* PLA kit (Sigma-Aldrich), according to the manufacturers’ protocol. After nuclei were stained using Hoechest (Thermo Fisher), cells were observed with a confocal laser microscope (Nikon, Tokyo, Japan).

### Immunocytochemistry microscopy

At 70% confluence, TPC1 cells were seeded on glass cover slips, cultured overnight in a 12-well plate, and treated with CDDP for 4, 8, 12 or 24 hours. After treatment of CDDP, TPC1 cells were fixed with 4% paraformaldehyde for 10 min, permeabilized with 0.1% Triton X-100 in PBS for 5 min, incubated with 5% BSA for 1 hour. The cells were subsequently incubated with primary antibodies at 4°C overnight, followed by washing four times with 0.05% Tween-20 in PBS and staining with the secondary antibody for 2 hours at room temperature. After nuclei were stained using Hoechest (Thermo Fisher), cells were observed with a confocal laser microscope (Nikon, Tokyo, Japan).

### Subcellular fractionation

TPC1 cells were treated with CDDP for 4, 8, 12 or 24 hours in the absence of serum. After cells harvest, cytoplasmic, and nuclear fractionation were separated by using NE-PER Nuclear and Cytoplasmic extraction reagents (Thermo Fisher), according to the manufacturer's instructions.

### Chromatin immunoprecipitation (ChIP) assay

ChIP assay was performed using the ChIP assay kit (Millipore, Temecula, CA, USA) according to the manufacturer's instructions. Briefly, 2 × 10^7^ cells were fixed with 1% formaldehyde (Sigma-Aldrich) at room temperature for 10 min, and then they were lysed on ice for 20 min. These lysed extracts were subjected to shearing by sonication. After centrifugation at 13,000 x g at 4°C for 30 min to remove insoluble material, the soluble chromatin was subjected to immunoprecipitation with anti-HA (abcam). The primer sequences used in ChIPs were as follows: promoter of *MUL1* (−1566 to −1283), 5’-AAG TTG CAC TGA GCC GAG ATC-3’ and 5’-CTG GGA TTA CAG GCG TGA GTC-3’.

### Luciferase assay

TPC1 cells were transfected using the Lipofectamine 2000 with FOXO3 WT-HA or FOXO3 TM-HA or *MUL1* reporter constructs. After transfection, TPC1 cells were plated onto a 96-well plate at a density of 4,000 cells/well. After overnight TPC1 cells attachment, cells were then treated with CDDP for 24 hours. Luciferase reporter assay was performed using the Luciferase Assay System (Promega, Madison, WI, USA) as the manufacturer's instructions.

### Detection of AKT ubiquitylation

AKT ubiquitylation assays were determined by Ni-NTA His pull-down assays. Ni-NTA pull-down ubiquitylation assay was performed as described previously [30]. Briefly, TPC1 cells were transfected with pcDNA3.1-Myr AKT-Myc/His together with each indicated plasmids and then washed with PBS, lysed in 200 μl of denaturing lysis buffer (50 mM Tris-HCl, pH 7.4, 0.5% SDS and 70 mM β-meraptoethanol, a protease inhibitor cocktail, and 10 μM MG132) by vortexing, and boiled for 20 min at 95°C. The lysates were diluted with 800 μl buffer A (50 mM NaH_2_PO_4_, 300 mM NaCl, 10 mM imidazole, pH 8.0, protease inhibitor cocktail, and 10 μM MG132) and incubated overnight with 40 μl Ni-NTA Agarose Beads (Invitrogen) at 4°C. Beads were washed three times with buffer B (50 mM NaH_2_PO_4_, 300 mM NaCl, 20 mM imidazole, pH 8.0), and bound proteins were eluted by boiling in a mixture of 5X SDS-PAGE gel loading buffer and buffer C (50 mM NaH_2_PO_4_, 300 mM NaCl, 250 mM imidazole, pH 8.0) (1:4). Then, Ubiquitylated AKT was detected with anti-HA antibody in Western blot.

### Measurement of intracellular ROS production

TPC1 and BHP10-3 cells were pre-treated with 10 mM NAC for 1 hour and then, CDDP was treated further 24 hours. After CDDP treatment, cells were treated with 10 μM hydroethidine (Thermo Fisher) for 30 min at 37°C. Fluorescence-stained cells (1×10^4^) were quantified using the BD FACSAria III instrument (BD Biosciences, San Diego, CA, USA).

### Statistical analyses

Data are expressed as means ± standard deviations (SD). Data were analyzed by ANOVA, followed by Student's *t*-test and Tukey–Kramer multiple comparisons method. *P* < 0.05 was considered statistically significant.

## SUPPLEMENTARY MATERIALS FIGURES



## References

[1] Manning BD, Toker A (2017). AKT/PKB signaling: navigating the network. Cell.

[2] Vivanco I, Sawyers CL (2002). The phosphatidylinositol 3-Kinase AKT pathway in human cancer. Nat Rev Cancer.

[3] Hay N (2005). The Akt-mTOR tango and its relevance to cancer. Cancer Cell.

[4] Fruman DA, Rommel C (2014). PI3K and cancer: lessons, challenges and opportunities. Nat Rev Drug Discov.

[5] Mayer IA, Arteaga CL (2016). The PI3K/AKT pathway as a target for cancer treatment. Annu Rev Med.

[6] Kim MS, Jeong EG, Yoo NJ, Lee SH (2008). Mutational analysis of oncogenic AKT E17K mutation in common solid cancers and acute leukaemias. Br J Cancer.

[7] Malanga D, Scrima M, De Marco C, Fabiani F, De Rosa N, De Gisi S, Malara N, Savino R, Rocco G, Chiappetta G, Franco R, Tirino V, Pirozzi G (2008). Activating E17K mutation in the gene encoding the protein kinase AKT1 in a subset of squamous cell carcinoma of the lung. Cell Cycle.

[8] Matter MS, Decaens T, Andersen JB, Thorgeirsson SS (2014). Targeting the mTOR pathway in hepatocellular carcinoma: current state and future trends. J Hepatol.

[9] Brognard J, Clark AS, Ni Y, Dennis PA (2001). Akt/protein kinase B is constitutively active in non-small cell lung cancer cells and promotes cellular survival and resistance to chemotherapy and radiation. Cancer Res.

[10] David O, Jett J, LeBeau H, Dy G, Hughes J, Friedman M, Brody AR (2004). Phospho-Akt overexpression in non-small cell lung cancer confers significant stage-independent survival disadvantage. Clin Cancer Res.

[11] Philp AJ, Campbell IG, Leet C, Vincan E, Rockman SP, Whitehead RH, Thomas RJ, Phillips WA (2001). The phosphatidylinositol 3’-kinase p85alpha gene is an oncogene in human ovarian and colon tumors. Cancer Res.

[12] Tanno S, Yanagawa N, Habiro A, Koizumi K, Nakano Y, Osanai M, Mizukami Y, Okumura T, Testa JR, Kohgo Y (2004). Serine/threonine kinase AKT is frequently activated in human bile duct cancer and is associated with increased radioresistance. Cancer Res.

[13] Schlieman MG, Fahy BN, Ramsamooj R, Beckett L, Bold RJ (2003). Incidence, mechanism and prognostic value of activated AKT in pancreas cancer. Br J Cancer.

[14] Yamamoto S, Tomita Y, Hoshida Y, Morooka T, Nagano H, Dono K, Umeshita K, Sakon M, Ishikawa O, Ohigashi H, Nakamori S, Monden M, Aozasa K (2004). Prognostic significance of activated Akt expression in pancreatic ductal adenocarcinoma. Clin Cancer Res.

[15] Eser S, Reiff N, Messer M, Seidler B, Gottschalk K, Dobler M, Hieber M, Arbeiter A, Klein S, Kong B, Michalski CW, Schlitter AM, Esposito I (2013). Selective requirement of PI3K/PDK1 signaling for Kras oncogene-driven pancreatic cell plasticity and cancer. Cancer Cell.

[16] Garcia Pedrero JM, Fernandez MP, Morgan RO, Herrero Zapatero A, Gonzalez MV, Suarez Nieto C, Rodrigo JP (2004). Annexin A1 down-regulation in head and neck cancer is associated with epithelial differentiation status. Am J Pathol.

[17] Lim J, Kim JH, Paeng JY, Kim MJ, Hong SD, Lee JI, Hong SP (2005). Prognostic value of activated Akt expression in oral squamous cell carcinoma. J Clin Pathol.

[18] Brazil DP, Hemmings BA (2001). Ten years of protein kinase B signalling: a hard Akt to follow. Trends Biochem Sci.

[19] Stebbing J, Lit LC, Zhang H, Darrington RS, Melaiu O, Rudraraju B, Giamas G (2014). Oncogene.

[20] Hafizi S, Ibraimi F, Dahlbäck B (2005). C1-TEN is a negative regulator of the Akt/PKB signal transduction pathway and inhibits cell survival, proliferation, and migration. FASEB J.

[21] Maira SM, Galetic I, Brazil DP, Kaech S, Ingley E, Thelen M, Hemmings BA (2001). Carboxyl-terminal modulator protein (CTMP), a negative regulator of PKB/Akt and v-Akt at the plasma membrane. Science.

[22] Du K, Herzig S, Kulkarni RN, Montminy M (2003). TRB3: a tribbles homolog that inhibits Akt/PKB activation by insulin in liver. Science.

[23] Paramio JM, Segrelles C, Ruiz S, Jorcano JL (2001). Inhibition of protein kinase B (PKB) and PKCzeta mediates keratin K10-induced cell cycle arrest. Mol Cell Biol.

[24] Gao T, Furnari F, Newton AC (2005). PHLPP: a phosphatase that directly dephosphorylates Akt, promotes apoptosis, and suppresses tumor growth. Mol Cell.

[25] Mani A, Gelmann EP (2005). The ubiquitin-proteasome pathway and its role in cancer. J Clin Oncol.

[26] Chan CH, Jo U, Kohrman A, Rezaeian AH, Chou PC, Logothetis C, Lin HK (2014). Posttranslational regulation of Akt in human cancer. Cell Biosci.

[27] Dickey CA, Koren J, Zhang YJ, Xu YF, Jinwal UK, Birnbaum MJ, Monks B, Sun M, Cheng JQ, Patterson C, Bailey RM, Dunmore J, Soresh S (2008). Akt and CHIP coregulate tau degradation through coordinated interactions. Proc Natl Acad Sci U S A.

[28] Xiang T, Ohashi A, Huang Y, Pandita TK, Ludwig T, Powell SN, Yang Q (2008). Negative regulation of AKT activation by BRCA1. Cancer Res.

[29] Suizu F, Hiramuki Y, Okumura F, Matsuda M, Okumura AJ, Hirata N, Narita M, Kohno T, Yokota J, Bohgaki M, Obuse C, Hatakeyama S, Obata T (2009). The E3 ligase TTC3 facilitates ubiquitination and degradation of phosphorylated Akt. Dev Cell.

[30] Bae S, Kim SY, Jung JH, Yoon Y, Cha HJ, Lee H, Kim K, Kim J, An IS, Kim J, Um HD, Park IC, Lee SJ (2012). Akt is negatively regulated by the MULAN E3 ligase. Cell Res.

[31] Kim SY, Kim HJ, Kang SU, Kim YE, Park JK, Shin YS, Kim YS, Lee K, Kim CH (2015). Non-thermal plasma induces AKT degradation through turn-on the MUL1 E3 ligase in head and neck cancer. Oncotarget.

[32] Fu Z, Tindall DJ (2008). FOXOs, cancer and regulation of apoptosis. Oncogene.

[33] Ho KK, Myatt SS, Lam EW (2008). Many forks in the path: cycling with FoxO. Oncogene.

[34] Lam EW, Brosens JJ, Gomes AR, Koo CY (2013). Forkhead box proteins: tuning forks for transcriptional harmony. Nat Rev Cancer.

[35] Coomans de Brachene A, Demoulin JB (2016). FOXO transcription factors in cancer development and therapy. Cell Moll Life Sci.

[36] Kikuno N, Shiina H, Urakami S, Kawamoto K, Hirata H, Tanaka Y, Place RF, Pookot D, Majid S, Igawa M, Dahiya R (2007). Knockdown of astrocyte-elevated gene-1 inhibits prostate cancer progression through upregulation of FOXO3a activity. Oncogene.

[37] de Keizer PL, Packer LM, Szypowska AA, Riedl-Polderman PE, van den Broek NJ, de Bruin A, Dansen TB, Marais R, Brenkman AB, Burgering BM (2010). Activation of forkhead box O transcription factors by oncogenic BRAF promotes p21cip1-dependent senescence. Cancer Res.

[38] Paik JH, Kollipara R, Chu G, Ji H, Xiao Y, Ding Z, Miao L, Tothova Z, Horner JW, Carrasco DR, Jiang S, Gilliland DG, Chin L (2007). FoxOs are lineage-restricted redundant tumor suppressors and regulate endothelial cell homeostasis. Cell.

[39] Brunet A, Bonni A, Zigmond MJ, Lin MZ, Juo P, Hu LS, Anderson MJ, Arden KC, Blenis J, Greenberg ME (1999). Akt promotes cell survival by phosphorylating and inhibiting a Forkhead transcription factor. Cell.

[40] Itoh T, Terazawa R, Kojima K, Nakane K, Deguchi T, Ando M, Tsukamasa Y, Ito M, Nozawa Y (2011). Cisplatin induces production of reactive oxygen species via NADPH oxidase activation in human prostate cancer cells. Free Radic Res.

[41] Riesterer O, Zingg D, Hummerjohann J, Bodis S, Pruschy M (2004). Degradation of PKB/Akt protein by inhibition of the VEGF receptor/mTOR pathway in endothelial cells. Oncogene.

[42] Ringel MD, Hayre N, Saito J, Saunier B, Schuppert F, Burch H, Bernet V, Burman KD, Kohn LD, Saji M (2001). Overexpression and overactivation of Akt in thyroid carcinoma. Cancer Res.

[43] Weidinger C, Krause K, Mueller K, Klagge A, Fuhrer D (2011). FOXO3 is inhibited by oncogenic PI3K/Akt signaling but can be reactivated by the NSAID sulindac sulfide. J Clin Endocrinol Metab.

[44] Calnan DR, Krause K, Mueller K, Klagge A, Fuhrer D (2008). FOXO3 is inhibited by oncogenic PI3K/Akt signaling but can be reactivated by the NSAID sulindac sulfide. Oncogene.

[45] Quinn MF (1989). Relation of observer agreement to accuracy according to a two-receiver signal detection model of diagnosis. Med Decis Making.

[46] Li Y, Yu J, D DU, Fu S, Chen Y, Yu F, Gao P (2013). Involvement of post-transcriptional regulation of FOXO1 by HuR in 5-FU-induced apoptosis in breast cancer cells. Oncol Lett.

[47] Sunters A, Fernández de Mattos S, Stahl M, Brosens JJ, Zoumpoulidou G, Saunders CA, Coffer PJ, Medema RH, Coombes RC, Lam EW (2003). FoxO3a transcriptional regulation of Bim controls apoptosis in paclitaxel-treated breast cancer cell lines. J Biol Chem.

[48] Essafi A, Fernández de Mattos S, Hassen YA, Soeiro I, Mufti GJ, Thomas NS, Medema RH, Lam EW (2005). Direct transcriptional regulation of Bim by FoxO3a mediates STI571-induced apoptosis in Bcr-Abl-expressing cells. Oncogene.

[49] Yang JY, Hung MC (2011). Deciphering the role of forkhead transcription factors in cancer therapy. Curr Drug Targets.

[50] Parbhu VV, Allen JE, Dicker DT, El-Deiry WS (2015). Small-molecule ONC201/TIC10 targets chemotherapy-resistant colorectal cancer stem-like cells in an Akt/Foxo3a/TRAIL-dependent manner. Cancer Res.

[51] Remandez de Mattos S, Villalonga P, Clardy J, Lam EW (2008). FOXO3a mediates the cytotoxic effects of cisplatin in colon cancer cells. Mol Cancer Ther.

[52] Cortes R, Tarrado-Castellarnau M, Talancón D, López C, Link W, Ruiz D, Centelles JJ, Quirante J, Cascante M (2014). A novel cyclometallated Pt(II)-ferrocene complex induces nuclear FOXO3a localization and apoptosis and synergizes with cisplatin to inhibit lung cancer cell proliferation. Metallomics.

[53] Liu H, Yin J, Wang C, Gu Y, Deng M, He Z (2014). FOXO3a mediates the cytotoxic effects of cisplatin in lung cancer cells. Anticancer Drugs.

[54] Tarrado-Castellarnau M, Cortés R, Zanuy M, Tarragó-Celada J, Polat IH, Hill R, Fan TW, Link W, Cascante M (2015). Methylseleninic acid promotes antitumour effects via nuclear FOXO3a translocation through Akt inhibition. Pharmacol Res.

[55] Fang L, Wang H, Zhou L, Yu D (2011). FOXO3a reactivation mediates the synergistic cytotoxic effects of rapamycin and cisplatin in oral squamous cell carcinoma cells. Toxicol Appl Pharmacol.

[56] Brown SJ, Kellett PJ, Lippard SJ (1993). Ixr1, a yeast protein that binds to platinated DNA and confers sensitivity to cisplatin. Science.

[57] Ohndorf UM, Rould MA, He Q, Pabo CO, Lippard SJ (1999). Basis for recognition of cisplatin-modified DNA by high-mobility-group proteins. Nature.

[58] Jamieson ER, Lippard SJ (1999). Structure, recognition, and processing of cisplatin-DNA adducts. Chem Rev.

