# Supplementary material for: FOXO3 induces ubiquitylation of AKT through MUL1 regulation
Source: Oncotarget. 2017 Nov 30;8(66):110474–89. doi: 10.18632/oncotarget.22793 (PMC5746397; doi:10.18632/oncotarget.22793)
Supplement: Supplementary file 1 [file oncotarget-08-110474-s001.pdf]

FOXO3 induces ubiquitylation of AKT through MUL1 regulation

SUPPLEMENTARY MATERIALS

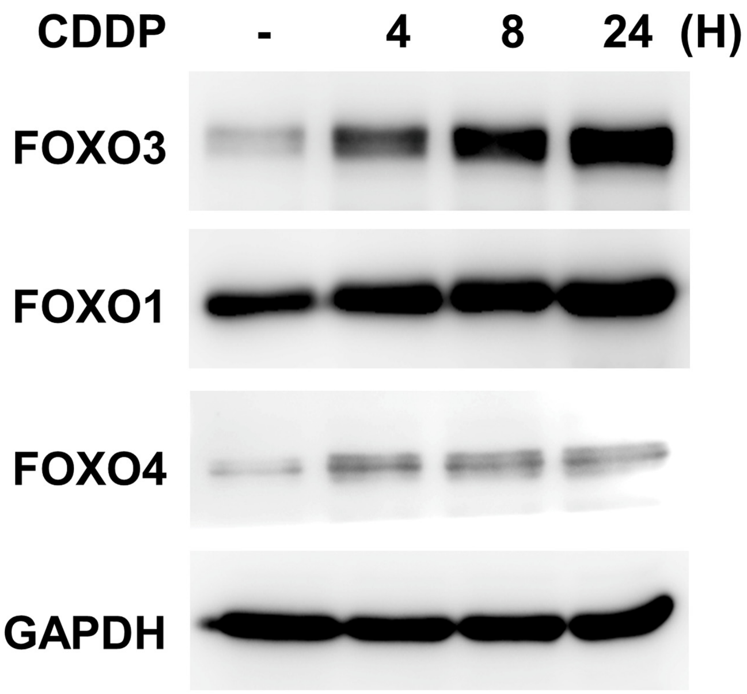

Supplementary Figure 1: FOXO3 is strongly accumulated by CDDP. CDDP (30 µM) was treated at TPC1 for the indicated times in the absence of serum. FOXO1, FOXO3 of FOXO4 levels were determined by Western blot assay.

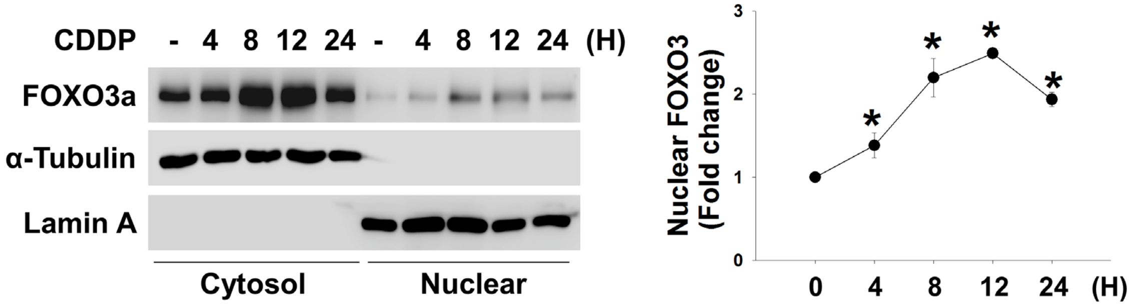

Supplementary Figure 2: CDDP induces thyroid cancer cell death in a time dependent fashion. CDDP was treated to TPC1 cells for the indicated times. After cellular fractionation, FOXO3 levels were performed by Western blot.

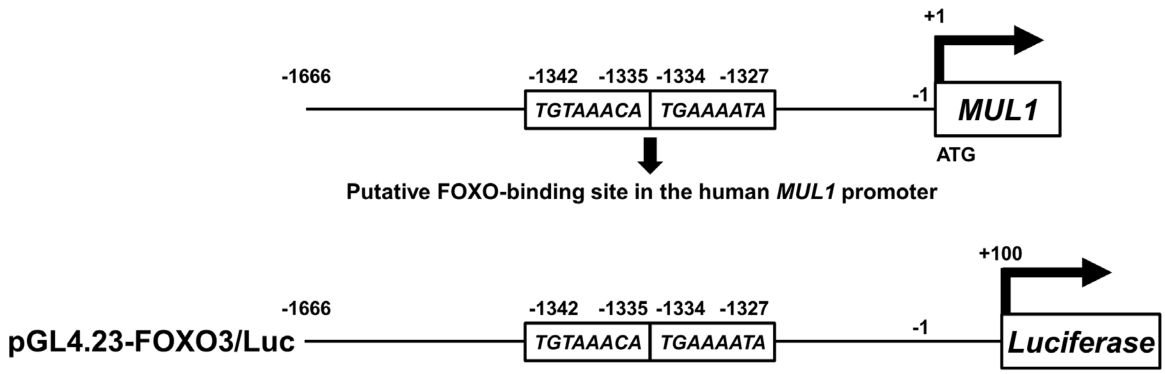

Supplementary Figure 3: Scheme of *MUL1* promoter region cloning for FOXO3 reporter assay.

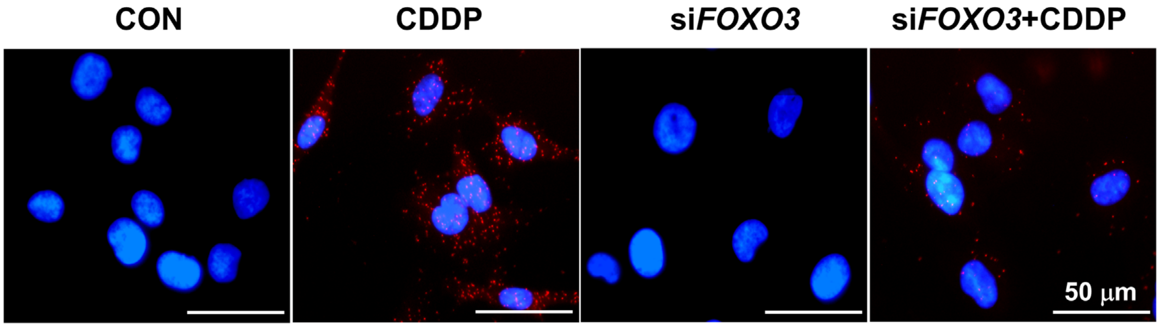

Supplementary Figure 4: FOXO3 knock-down inhibits CDDP-induced interaction between MUL1 and AKT. Scrambled RNAs or *FOXO3* siRNA transfected TPC1 cells were grown onto coverslip in a 12-well plate and then, CDDP was treated for 12 hours. The interaction between AKT and MUL1 was determined by PLA assay. Fluorescence images were observed by confocal microscopy. Scare bar represents 50 μm.
